# Enhanced Stability of Long-Living Immobilized Recombinant β-d-*N*-Acetyl-Hexosaminidase A on Polylactic Acid (PLA) Films for Potential Biomedical Applications

**DOI:** 10.3390/jfb12020032

**Published:** 2021-05-11

**Authors:** Eleonora Calzoni, Alessio Cesaretti, Nicolò Montegiove, Alessandro Di Michele, Carla Emiliani

**Affiliations:** 1 Department of Chemistry, Biology and Biotechnology, University of Perugia, 06123 Perugia, Italy; eleonoracalzoni@gmail.com (E.C.); nicolo.montegiove@gmail.com (N.M.); carla.emiliani@unipg.it (C.E.); 2 Center of Excellence on Innovative Nanostructured Materials—CEMIN, University of Perugia, 06123 Perugia, Italy; 3 Department of Physics and Geology, University of Perugia, 06123 Perugia, Italy; alessandro.dimichele@unipg.it

**Keywords:** enzyme immobilization, biopolymers, enzyme stability, enzyme recyclability, biocatalysis, lysosomal storage diseases, enzymatic replacement therapy

## Abstract

β-d-*N*-acetyl-hexosaminidase (Hex, EC 3.2.1.52) is an acid hydrolase that catalyzes the cleavage of the β-1,4 bond in *N*-acetyl-d-galactosamine (Gal-NAc) and *N*-acetyl-d-glucosamine (Glc-NAc) from the non-reducing end of oligosaccharides and glycoconjugates. It is widely expressed in both the prokaryotic and eukaryotic world, where it performs multiple and important functions. Hex has antifungal activity in plants, is capable of degrading many biological substrates, and can play an important role in the biomedical field for the treatment of Tay-Sachs and Sandhoff diseases. With the aim being able to obtain a device with a stable enzyme, a method of covalent immobilization on polylactic acid (PLA) films was developed for the A isoform of the β-d-*N*-acetyl-hexosaminidase enzyme (HexA), produced in a recombinant way from Human Embryonic Kidney-293 (HEK-293) cells and suitably purified. An in-depth biochemical characterization of the immobilized enzyme was carried out, evaluating the optimal temperature, thermal stability, pH parameters, and Km value. Moreover, the stability of the enzymatic activity over time was assessed. The results obtained showed an improvement in terms of kinetic parameters and stability to heat for the enzyme following immobilization and the presence of HexA in two distinct immobilized forms, with an unexpected ability for one of them to maintain its functionality for a long period of time (over a year). The stability and functionality of the enzyme in its immobilized form are therefore extremely promising for potential biotechnological and biomedical applications.

## 1. Introduction

β-d-*N*-acetyl-hexosaminidase (Hex, EC 3.2.1.52) is an acid hydrolase whose main function is to catalyze the cleavage of the β-1,4 bond in *N*-acetyl-d-galactosamine (Gal-NAc) and *N*-acetyl-d-glucosamine (Glc-NAc) from the non-reducing end of oligosaccharides and glycoconjugates, such as glycoproteins, glycolipids, and glycosaminoglycans (GAGs) [1,2,3]. The β-d-*N*-acetyl-hexosaminidase enzyme has been isolated in many living organisms, from prokaryotes to eukaryotes, where it performs different functions depending on the cellular location and the organism of origin [3]. In the eukaryotic domain, the animal Hex is certainly the most studied form and its localization is purely lysosomal; while in prokaryotes, some studies have highlighted its presence in the periplasmic membrane and the cytoplasmic granules [3,4,5]. This enzyme is particularly interesting from a biotechnological point of view as it takes part in the chitin degradation cascade, affecting the growth of the fungal and bacterial cell wall, and acts as a powerful defense mechanism in plants in response to fungal infections [3,6,7,8,9]. Further to this, specific newly discovered forms of the Hex enzymes encoded by some bacteria were found to serve a role in the production of human milk oligosaccharide precursors [10,11], thus paving the way for other viable industrial applications for this family of enzymes.

The role of β-d-*N*-acetyl-hexosaminidase is, however, mainly studied in humans, where there are two main isoforms of this lysosomal enzyme, called β-d-*N*-acetyl-hexosaminidase A (HexA) and β-d-*N*-acetyl-hexosaminidase B (Hex B) [12]. HexA is a heterodimer made up of α (60 kDa) and β (65 kDa) subunits, HexB is instead a ββ homodimer. There is also a third isoenzymatic form, consisting of two α subunits, called HexS (αα), which is not detectable in normal human tissues, since it is unstable and synthesized in inconsistent quantities; however, it represents the main form in Sandhoff disease and some types of leukemia [13,14,15,16]. Starting from the synthesis of the pro-polypeptides of the α and β subunits, post-translational processes occur in the lysosome to form mature enzymes: the pro-α-polypeptide is cleaved into two chains of 6 and 54 kDa, while the pro-β-polypeptide is broken down into three chains: propeptide (7–10 kDa), β_a_ (30 kDa), and β_b_ (24–26 kDa). In both cases, the polypeptides are held together by disulfide bonds [17]. The α and β subunits have a 60% similarity as for their primary structure; consequently, the two polypeptide chains exhibit very similar three-dimensional structures with conserved domains. Both subunits possess active sites, but the presence of an arginine in position 424 in the α subunit (αArg424) allows the binding of substrates containing 6-sulfate, while the presence of an aspartate in position 452 in the β subunit (βAsp452) hinders the formation of this link [18]. As a consequence, all the three enzymes are able to break the β-1,4 glycosidic bonds present in substrates such as oligosaccharides or glycoconjugates; however, only the enzymes containing the α subunit (HexA and HexS), with a positively charged amino acid residue at its binding site, can hydrolyze the β-1,4 bonds of GlcNAc-6-sulfate residues, and only HexA, which is a heterodimer, is able to cleave the β-1,4 Gal-NAc bond of sialic acid present in the GM2 ganglioside [12,19,20].

The α-subunit of β-d-*N*-acetyl-hexosaminidase is encoded by the HEXA gene, located in position 23–24 on the long arm of chromosome 15 (15q23-24). The HEXB gene, on the other hand, codes for the β-subunit of the enzyme and is located on the long arm of chromosome 5 (5q13). Mutations affecting the HEXA or HEXB genes result in serious Lysosomal Storage Diseases (LSDs), genetic disorders causing the metabolic accumulation of natural substrates, particularly the GM2 ganglioside, which, reaching toxic levels, leads to the death of the patient. In particular, mutations of the gene that codes for the α-subunit cause Tay-Sachs disease or B variant whereas mutations of the gene that codes for the β-subunit cause the onset of Sandhoff disease or 0 variant [21]. These pathologies are clinically similar and share the accumulation of gangliosides, globosides, and non-degraded glycolipids. The accumulation of gangliosides mainly occurs in neurons, while the other substrates are primarily found in the viscera [22,23,24,25,26,27,28,29]. Over the past few years, some therapeutic approaches have been tested, but they have often given unsatisfactory results for both Tay-Sachs and Sandhoff diseases [23,30,31]. Different strategies have been tested to alleviate the symptoms of these pathologies [32,33], such as gene therapy (GT) [19], the use of molecular chaperones [34], and Enzyme Replacement Therapy (ERT) [35,36]. Although ERT represents the most promising strategy, it still has limitations due to the difficulty of the recombinant enzyme in crossing the Blood–Brain Barrier (BBB) [30,31,37,38]. Therefore, some studies are currently underway aiming at providing recombinant enzymes capable of passing through the BBB [31,39,40,41].

There are many applications where β-d-*N*-acetyl-hexosaminidase can be used due to its multiple functions. Therefore, it is necessary to improve the stability and handling of the enzyme in order to create an effective device that can be used for biotechnological applications, in the industry and especially in the biomedical field (i.e., treatment of Tay-Sachs and Sandhoff diseases).

To this purpose, enzyme immobilization is an extremely interesting and promising technique recently employed in biotechnological industries, where enzymes are used for different purposes. It consists of the binding of free enzymes on different types of inert supports, the aim being to improve their stability and recyclability [42,43,44,45]. The use of immobilized enzymes creates practical, economic, and ecological advantages. Their use grants a simpler and more effective manipulation, as they are found in solid formulations rather than in liquid ones, allowing the complete recovery of the enzyme. The conservation of the catalytic activity is the main feature that has to be maintained during the process; hence, it is important not to alter the conformation and the structure of the macromolecule. Based on the chemical and physical properties of both the enzyme and the support, the most suitable immobilization technique can be chosen [42,46]. The enzyme industry is constantly growing and is longingly looking for more sustainable industrial processes that allow higher yields and greater efficiency. To date, there are very many industrial sectors that resort to immobilized enzymes, ranging from food to textile and detergent industry, not to mention the production of biofuels. Other important applications also concern the pharmaceutical and biomedical industries, where enzymes are used for therapeutic purposes [47,48,49]. One of the main goals achieved through enzymatic immobilization in the medical field is the treatment of those pathologies caused by enzymatic deficits that provoke serious neurological damage, as is the case with LSDs caused by mutations of genes encoding key enzymes of the lysosomal compartment. In this scenario, the success of the therapy is conditioned by the actual possibility to reach the central nervous system (CNS). Enzymes immobilized on nanoparticles represent a viable strategy in this sense, as these nanometric bodies possess the proper dimension to cross biological membranes, plus their surface can be functionalized in order to enhance their receptor-mediated transport across the BBB [39,50,51,52,53,54].

In this light, the aim of our work is to develop an immobilized system for the β-d-*N*-acetyl-hexosaminidase enzyme. In this study, HexA was expressly chosen to be tested as it represents the most important isoform from the biomedical point of view, inasmuch as the HEXA gene mutation is involved in both Tay-Sachs and Sandhoff diseases, contrarily to HexB, which is only involved in Sandhoff disease [21]. The HexA enzyme was produced in a recombinant way and purified from HEK-293 cells overexpressing the α-subunit, and covalently immobilized on polymeric films, with the aim of obtaining a stabilized biocatalyst that could be tested for potential biotechnological applications, especially in the biomedical field. In fact, the HexA enzyme is rather stable in solution, but the possibilities of exploiting its functions in its free forms are rather limited in that free enzymes are found in solution together with the products of their reaction, so that they cannot be reused unless they undergo expensive and time-consuming purification procedures. Conversely, the great advantage of resorting to immobilized enzymes comes from the fact that the latter are in a different phase relative to the products, which can be easily removed, as such. Moreover, immobilization on a polymeric scaffold allows the solid support to be shaped and specifically designed depending on the final function it might serve. The polymer of choice for this purpose was polylactic acid (PLA) because, on the one hand, it is a biocompatible and biodegradable material, with an average half-life of 30 weeks when in contact with biological media [55], and on the other hand, it can be molded into practically any shape (e.g., films, nanoparticles, and columns), being thus suitable for both biomedical and industrial applications [47,56].

## 2. Materials and Methods

### 2.1. Materials

Polylactic acid (PLA), Diethylentriamine, and 25% Glutaraldehyde stock solution were purchased from Sigma-Aldrich. 4-methylumbelliferyl-*N*-acetyl-β-d-glucosaminide (MUG) from Sigma-Aldrich (Saint Louis, MO, USA), while 4-methylumbelliferyl-6-sulfo-2-acetamido-2-deoxy-β-d-glucopyranoside (MUGS) from Toronto Chemicals (Toronto, ON, Canada). Dulbecco’s modified Eagle’s medium (DMEM), fetal bovine serum (FBS), Trypsin, G418 Sulphate (Geneticin Solution 50 mg/mL), and Penicillin/Streptomycin were purchased from Euroclone (Pero, Italy). The restriction enzymes were purchased from New England Biolabs (Ipswich, MA, USA). SuperScript II Reverse Transcriptase and lipofectamine were purchased from Invitrogen (Carlsbad, CA, USA). DE-52 DEAE-cellulose was purchased from Whatman Ltd. (Maidstone, Kent, UK) and Concanavalin A-Sepharose from Sigma-Aldrich (Saint Louis, MO, USA). For SDS-Page Electrophoresis, 30% acrylamide/bis-acrylamide solution was purchased from Bio-Rad (Hercules, CA, USA), Trizma base, Glycine reagent, N,N,N′,N′-Tetramethylethylenediamine, Ammonium persulfate, Dithiothreitol (DTT), and sodium dodecyl sulfate (SDS) from Sigma-Aldrich (Saint Louis, MO, USA), and Prestained Protein SHARPMASS™ VI protein MW marker from Euroclone (Pero, Italy). For immunoblot analysis, polyvinylidene difluoride (PVDF) membrane (Thermo Fisher Scientific, Waltham, MA, USA), Invitrogen HexA polyclonal antibody (Thermo Fisher Scientific, Waltham, MA, USA), Anti-rabbit IgG, HRP-linked antibody (Cell Signaling Technology, Danvers, MA, USA), and Pierce™ ECL Western Blotting Substrate (Thermo Fisher Scientific, Waltham, MA, USA).

### 2.2. HEK-293 Cell Culture

Human Embryonic Kidney-293 (HEK-293) cells (ATCC, Manassas, VA, USA) were cultured in a DMEM medium containing 10% (*v*/*v*) heat-inactivated FBS and Penicillin 10,000 U per mL/Streptomycin 10 mg per mL. Cell viability, determined by Trypan blue, under the differential experimental conditions was about 95%.

### 2.3. HEK-293 Cell Line Transfection

The cDNA of the human Hex α-subunit was cloned into a pUC19 plasmid. The plasmid pUC19 lacked a short sequence at the 3′ end of the α-cDNA; therefore, the restriction fragment containing the sequence 3′ of the missing α-cDNA was amplified, using a forward primer (5′-TGTGGACAACACAAACCTGG-3′) and a reverse primer (5′-TCTACGTCTAGAGCGGCCGCTTCAGGTCTGTTCAAACT-3′). Subsequently, the restriction fragment was cloned in the plasmid using the HincII and XbaI restriction sites, to obtain the plasmid pUC19 with the complete sequence of α-cDNA. α-cDNA was then cloned into the pcDNA3 vector using the HindIII and XbaI restriction sites. HEK-293 cells were transfected with the pcDNA3 vector lacking α-cDNA and with the recombinant pcDNA3 vector using a standard method with Lipofectamine, a molecule that binds the DNA to be transfected and facilitates its entry into the cells. The transfected clones overexpressing HexA (HEK-HexA) were selected and kept in culture in the same medium containing 800 µg/mL of antibiotic Geneticin (G418).

### 2.4. Preparation of Cell Lysates

Cell samples were washed twice with phosphate buffer saline (PBS) and suspended in 10 mM sodium phosphate buffer (pH 6.0) and 0.05% (*v*/*v*) Triton X-100 detergent. After 1 h of incubation in ice, they were harvested, sonicated using a VirTis Virsonic 100 Ultrasonic Cell Disrupter (SP Industries, Warminster, PA, USA)—four sonication runs, 15 s each), then centrifuged (5804 R, Eppendorf, Hamburg, Germany) at 16,000× *g* for 15 min. All procedures were carried out at 4 °C. The supernatants containing cell lysates were used for the subsequent analyses.

### 2.5. Determination of Protein Concentration by Bradford Method

The protein content was determined by the Bradford method [57], using Quick Start™ Bradford 1× Dye Reagent (Bio-Rad, Hercules, CA, USA) according to the manufacturer’s instructions for one-step determination of protein concentration. The quantitative determination was carried out using the Coomassie Brilliant Blue G-250 dye (Bio-Rad, Hercules, CA, USA), which in the protein-bound form has an absorption peak at 595 nm. The absorbance at 595 nm was measured using a Shimadzu UV-160A UV-Visible Recording Spectrophotometer (Shimadzu Scientific Instruments, Kyoto, Japan). The concentration of the soluble proteins of the samples was obtained from their absorbance using a calibration curve prepared with known concentrations of bovine serum albumin (BSA; Sigma-Aldrich, Saint Louis, MO, USA).

### 2.6. β-d-N-Acetyl-Hexosaminidase Enzyme Assay

Levels of activity of the β-d-*N*-acetyl-hexosaminidase were measured using 3 mM solutions of the specific synthetic fluorogenic substrates 4-methylumbelliferyl-β-*N*-acetylglucosaminide (MUG) and 4-methylumbelliferyl-β-*N*-acetylglucosaminide-6-sulfate (MUGS), dissolved in 0.1 M citrate/0.2 M phosphate buffer, pH 4.5, in 96-well black polystyrene microplates (Greiner Bio-One GmbH, Frickenhausen, Germany). At the end of the reaction time, 0.290 mL of 0.4 M glycine/NaOH buffer, pH 10.4, were added. Fluorescence of the released 4-methylumbelliferone was measured by means of an Infinite F200 fluorescence microplate reader (Tecan Group Ltd., Männedorf, Switzerland) at 360 nm excitation, 450 nm emission. The milliunits (mU) of enzyme, where 1 mU is the amount of enzyme that hydrolyses 1 nmol of substrate/min at 37 °C, were calculated by referring to a calibration curve set up with 4-methylumbelliferone at different concentrations. Specific activity was expressed as enzyme mU/mg of proteins.

### 2.7. Affinity Chromatography on Concanavalin A-Sepharose

The partial purification of HexA was obtained by affinity chromatography on Concanavalin A-Sepharose with a protocol previously developed in our laboratory [58]. Lysates of HEK-HexA cells were dialyzed overnight against 20 mM Tris/HCl buffer, pH 7.4, containing 1 mM MnCl_2_, 1 mM MgCl_2_, and 1 mM CaCl_2_, and loaded onto a 5 mL Concanavalin A-Sepharose column equilibrated with the same buffer. The column was eluted with this buffer until the absorbance at 280 nm was zero. Glycoproteins retained by the column were eluted as a single peak of activity with a linear gradient of 0–0.5 M methyl α-mannoside in 50 mM Tris/HCl buffer, pH 7.4, containing 1 M NaCl (100 mL).

### 2.8. DEAE-Chromatography

Separation and analysis of Hex isoenzymes from the peak of Concanavalin A-Sepharose chromatography was performed by ion-exchange chromatography on DEAE-cellulose. DEAE-cellulose chromatography was performed as described for the first time by Robinson and Stirling (1968) [59] with a 2.5 mL column equilibrated with 10 mM sodium phosphate buffer (Na/P), pH 6.0, and loaded with 2 mg of total proteins. Enzyme molecules bound to the column were eluted in a linear gradient of NaCl that reached a concentration of 0.4 M in a 40 mL buffer. Fractions of 1 mL each were collected, and the activity was measured on each of them with the MUG and MUGS substrates as previously described. The fractions assigned to the HexA peak were combined together, and an average HexA concentration of about 0.10 mg/mL was assessed by performing the Bradford assay.

### 2.9. SDS-PAGE and Immunoblot Analysis

The HexA fractions resulting from DEAE-Chromatography were subjected to 10% SDS-PAGE (Mini-PROTEAN^®^ 3 Cell, Bio-Rad, Hercules, CA, USA) under reducing conditions according to Laemmli’s method [60]. The proteins were transferred to a polyvinylidene difluoride (PVDF) membrane using the Trans-Blot Turbo Transfer System (Bio-Rad, Hercules, CA, USA). The blotted PVDF membrane was washed three times in PBS with 0.1% Tween 20 detergent (*v*/*v*). The membrane was later incubated in blocking solution (5% milk powder (*w*/*v*) in PBS buffer + 0.1% (*v*/*v*) Tween 20) for 1 h at room temperature and then incubated over/night with an anti-α-subunit primary antibody diluted 1:1000 in a PBS solution containing 5% BSA (*w*/*v*) and 0.1% Tween 20 (*v*/*v*). After 3 washes in PBS + 0.1% (*v*/*v*) Tween 20, the membrane was incubated for 1 h at room temperature in a PBS solution containing 1% BSA (*w*/*v*) and 0.1% Tween 20 (*v*/*v*) with the secondary antibody conjugated with horseradish peroxidase (HRP). Blots were analyzed by the ECL (Enhanced Chemiluminescence) detection system.

### 2.10. Immobilization of β-d-N-Acetyl-Hexosaminidase A

The PLA powder was dissolved in chloroform at a concentration of 5% (*w*/*v*). Part of the solution was deposited on a Teflon disk placed in the rotor of a centrifuge (5804 R, Eppendorf, Hamburg, Germany) at 775× *g* for one minute. The PLA films were then dried for 48 h at room temperature, removed from the Teflon disk, and finally cut into pieces with a surface of about 1 cm^2^ (1 cm × 1 cm). The enzyme immobilization protocol involves a phase of functionalization of the support material and a subsequent activation phase [61]. A diethylenetriamine solution diluted in propanol at a concentration of 21% (*v*/*v*) was used to react with the material for 1 h at 55 °C. By doing so, it was possible to functionalize the polymer with NH_2_ groups. Subsequently, the PLA films were activated with a 2.5% glutaraldehyde solution in deionized H_2_O for 3 h at room temperature. Glutaraldehyde is a bifunctional agent as it can bind, with its two aldehydic groups, to both the NH_2_ groups of the functionalized material and to the NH_2_ groups of the side chains of the amino acids that make up the enzyme. After removal of the glutaraldehyde solution and abundant washing of the PLA films with deionized H_2_O, the previously purified HexA enzyme was immobilized, leaving 500 µL of the 50 µg/mL enzymatic solution in an overnight incubation at 4 °C. After the immobilization, several washes were carried out to remove non- and loosely bound enzymatic molecules, as long as no activity was detected in the washing solution. The washes were carried out under the same conditions of time, temperature, and shaking as the enzymatic activity assay to be sure that no further detachment would later be induced. The determination of the HexA concentration linked to the polymer was determined by the difference between the protein concentration determined by the Bradford method performed on the purified enzyme stock solution and that of the solution recovered after immobilization. The HexA-PLA preparations were stored in 10 mM sodium phosphate buffer (Na/P), pH 6.0 at 4 °C, without needing any additive.

### 2.11. Biochemical Characterization of Immobilized β-d-N-Acetyl-Hexosaminidase A

The activity of immobilized HexA was evaluated by performing the enzymatic assay with the MUG and MUGS substrates under the same reaction conditions described in paragraph 2.6. The substrate solutions were placed in contact with the HexA-PLA films, incubated at 37 °C for the desired time, and then moved in a 96-well black polystyrene microplate (Greiner Bio-One GmbH, Frickenhausen, Germany), where 0.290 mL of 0.4 M glycine/NaOH buffer, pH 10.4 were finally added. The fluorescence intensity was thus related to milliunits (mU) of enzyme per unit of area, giving the enzymatic activity in mU/cm^2^.

Stability towards pH was tested using the MUG substrate in a pH range between 2 and 9. In particular, 0.1 M citric acid was used to reach pH 2 and a 0.2 M sodium phosphate solution to attain pH 9; citrate/phosphate buffer solutions have instead been employed to achieve any desired intermediate pH values. For each pH, the reaction was conducted at 37 °C for 15 min. At the end of the reaction, the fluorescence intensity of the hydrolyzed substrate was measured and the level of enzymatic activity was subsequently derived.

The Km values of free and immobilized enzymes were determined using the linear transformation of Lineweaver and Burk. The samples were incubated with the artificial MUG substrate in a 0.06–2.0 mM concentration range for 15 min at 37 °C. Km values are expressed as concentration units (mM).

The thermal stability of both free and immobilized HexA enzymes was tested as a function of time by bringing the enzyme to the temperature of interest and waiting for increasing times. The activity towards MUG was later studied at 37 °C, revealing whether different temperatures had caused any loss of activity. Finally, the temperature optimum of free and immobilized HexA was evaluated by using the MUG substrate in a temperature range going from 4 °C to 80 °C. In particular, the temperature effect on the free enzyme was studied by adding the enzyme to the substrate at the desired temperature and incubated for 15 min. Whereas, as far as the immobilized enzyme is concerned, the HexA-PLA preparations were brought to the temperature of interest and after waiting 30 min to equilibrate, they were incubated with the artificial substrate for the usual 15 min. At the end of the reaction, the fluorescence intensity of the hydrolyzed substrate was recorded in order to determine the enzymatic activity.

Preliminary tests showed there is a linear relation between product release and time, at least up to 30 min, under all the experimental conditions employed, allowing fluorescence intensity to be quantitatively correlated with the mU of enzymatic activity in all cases. Fluorescence intensity was always detected using an Infinite F200 fluorescence microplate reader (Tecan Group Ltd., Männedorf, Switzerland) at 360 nm excitation, 450 nm emission.

## 3. Results and Discussion

### 3.1. HexA Purification by Affinity and DEAE Chromatography

**Figure 1:**
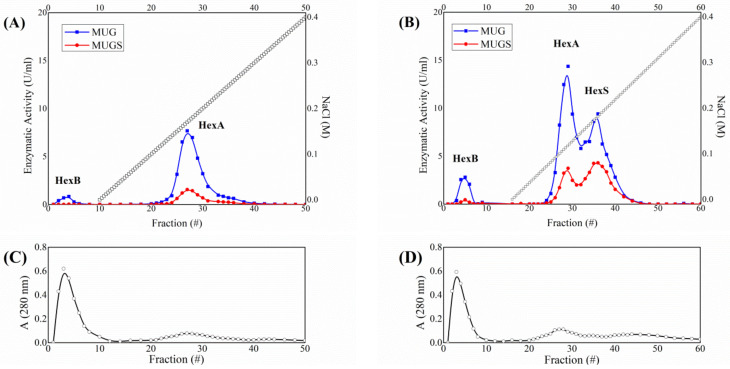
Analysis of Hex isoenzyme pattern on CTRL (**A**) and HEK-HexA cells (**B**). Hex isoenzyme separation was performed by DEAE-cellulose ion-exchange chromatography. Fractions of 1 mL in volume were collected and assayed for Hex activity using the fluorescent substrates MUG, which is hydrolyzed by both the α- and β-subunits forming Hex isoenzymes, and MUGS, which is only hydrolyzed by the α-subunit-containing isoforms. (**C**,**D**) Panels show the protein profile obtained by recording the absorbance of the collected fractions at 280 nm.

The purification of the enzyme was performed by two combined chromatographic separations: affinity chromatography on Concanavalin A-Sepharose followed by DEAE-cellulose ion-exchange chromatography. The initial lysate of overexpressing HEK-HexA cells exhibited a specific activity against the artificial substrate MUG of 42.5 mU/mg. After a first affinity chromatography, which eliminates those proteins which have no affinity for the resin, a marked increase in the specific activity is reported: the recorded value is 185 mU/mg. The separation and analysis of the isoenzymatic forms of Hex were then carried out by ion-exchange chromatography on DEAE-cellulose [62]. The combination of chromatographic separation and enzymatic activity assay with MUG and MUGS allows information about the subunit composition of the Hex isoforms expressed in the cells to be obtained [13]. In every chromatographic experiment, 2 mg of total proteins would be loaded into the column and fractions of 1 mL in volume would be collected and tested for Hex activity using the fluorogenic substrates MUG, which is hydrolyzed by both the α and β subunits, and MUGS, which is only hydrolyzed by the α subunits. Figure 1 shows two representative examples of the chromatographic profile of CTRL HEK cells and overexpressing HEK-HexA cells. Under the experimental conditions employed to perform DEAE-cellulose chromatographic analysis, HexB (ββ-homodimer) is not retained by the column and eluted with the void volume, whereas HexA (αβ-heterodimer) and HexS (αα-homodimer) are eluted by a linear saline gradient. The assignment of the chromatographic peaks to the different Hex isoforms can be carried out based on the ratio between the activity expressed against the two substrates, MUG and MUGS. The peak readily eluted with the buffer, is assigned to HexB, by virtue of its lack of activity against MUGS. As for the two peaks eluted with the saline gradient, the first exhibits a higher MUG/MUGS ratio, as expected for HexA, whereas the latter is characterized by a ratio close to 2, in agreement with its HexS assignment [13]. While the HexS peak can only be observed as a shoulder in the CTRL cell profile, it becomes particularly important for HEK-HexA cells, because of the overexpression of the α subunit, which allows the formation of the otherwise rare αα-homodimer. As for HEK-HexA cells, starting from a MUG/MUGS activity ratio of 2.6, the separation allows obtaining the HexA peak characterized by a ratio of 3.9 and a HexS peak which features an activity ratio of 1.9. The fractions assigned to the HexA peak after each chromatography were combined together, allowing an average HexA concentration of about 0.10 mg/mL to be achieved corresponding to about 0.4 mg of purified HexA. The specific activity concerning the HexA peak hits the value of 1250 mU/mg, which is the sign of a high purification grade (approaching a purification ratio value of 30 relatively to the crude extract) for the enzyme (Table 1).

### 3.2. SDS-PAGE and Immunoblot Analysis

**Figure 2:**
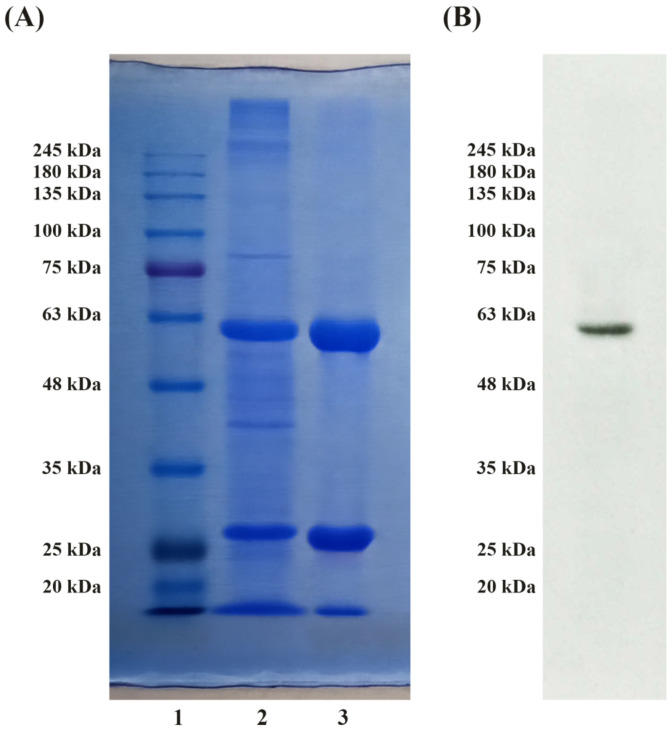
(**A**) SDS-PAGE followed by Coomassie Blue staining: molecular weight standards (lane 1); HEK-HexA crude extract (lane 2); purified HexA (lane 3). (**B**) Immunoblot analysis of purified HexA. The band placed between 63 kDa and 48 kDa corresponds to the α-subunit of the HexA enzyme.

The degree of HexA purification obtained by chromatography techniques was tested by polyacrylamide gel electrophoresis (SDS-PAGE) followed by Coomassie Brilliant Blue staining (Figure 2A); 20 μg of the crude extract and HexA peak were loaded in 10% SDS-PAGE gel. The correct degree of protein processing was also evaluated by Western Blotting. The molecular weight of the α-subunit of HexA (ca. 60 kDa) was detected by the polyclonal antibody for the α-subunit (Figure 2B).

The SDS-PAGE analysis followed by the Coomassie Blue staining showed two bands associated with the main polypeptide chains of the α and β (i.e., β_a_ and β_b_) subunits of the HexA heterodimer (Figure 2A). In fact, the presence of DTT breaks the disulfide bonds holding the chains in the two subunits, allowing the cleaved fragments of the polypeptides to be revealed. By comparing the intensities of the two bands for the purified enzyme with respect to the crude extract, third and second lanes of the gel, respectively, a sizeable enrichment of the HexA bands can be detected, revealing how the chromatographic process allowed a satisfactory purification of the enzyme to be obtained. This result is in agreement with the specific activity values previously measured, which are consistent with a high degree of purification. Immunoblot analysis also demonstrated the correct processing of HexA within the over-expressing cells, by revealing a net band placed below 63 kDa and corresponding to the main chain of the α-subunit of the HexA enzyme (Figure 2B).

### 3.3. HexA Immobilization on PLA Films

Purified HexA was then immobilized on PLA film preparations. The immobilization of the enzyme on PLA films was verified by the Bradford method and an average 65% immobilization yield was recorded, implying a concentration of 16 µg/cm^2^ for the immobilized enzyme. The activity of the immobilized molecules was then tested against both artificial substrates, MUG and MUGS. In both cases, the initial enzymatic activity was found to be significant (3.2 mU/cm^2^ and 2.0 mU/cm^2^ towards MUG and MUGS, respectively), but interestingly, the ratio between the substrates, which is 3.9 when HexA is in solution, is reduced to a value of about 1.6. This might be due to the link of the enzyme to the support, which could modify the active site conformations on the two subunits, thus altering the enzymatic activity towards the two substrates in a specific way. Specifically, a MUG/MUGS ratio of about 1.6, measured right after the immobilization, reveals a greater activity gained through immobilization for the α subunit (the only active against MUGS) relative to the β subunit, with respect to their behavior in solution.

**Figure 3:**
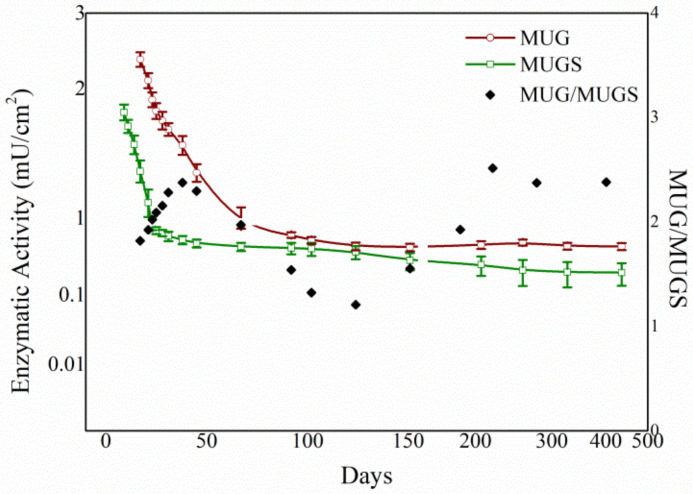
Trend of enzyme activity of HexA immobilized on PLA film against the two artificial substrates MUG and MUGS as a function of time, and variation of the activity ratio toward the two substrates (MUG/MUGS).

The enzymatic assay was repeated under the same conditions after immobilization on the different PLA film preparations in order to assess the stability over time of the activity of the HexA enzyme in its immobilized form. The evaluations were performed for more than one year after the immobilization event and the results shown in Figure 3 are the mean values of three HexA-PLA preparations. In the first weeks, the activity was tested every second day in order to monitor the decay trend recorded in this period. When the activity finally reached an almost constant value, the stability of the enzyme was tested less frequently, just to check whether the enzyme was keeping its catalytic ability.

The stability of enzymatic activity over time proved quite remarkable: following an initial decrease, supposedly due to a partial loss of activity of covalently bound molecules, a constant and stable enzymatic activity has been recorded for immobilized HexA for a period of time that, as of today, has exceeded one year. However, the ratio of MUG/MUGS activity was found to vary with the aging of the films (black rhombi in Figure 3), supposedly as a result of the different stability over time of the two subunits. A deeper insight into this behavior will be provided in Section 3.4. Interestingly enough, the preparations have been stored in 10 mM sodium phosphate buffer (Na/P), pH 6.0 at 4 °C, without needing any additive to avoid bacterial or fungal contamination of the films and preserve the enzymatic activity of immobilized HexA. This is confidently due to the antifungal and antibacterial activity expressed by the HexA molecule [7,9], which makes this enzyme also particularly appealing for different biotechnological applications other than the one in the biomedical field.

### 3.4. Biochemical Characterization of the Free and Immobilized HexA Enzyme on PLA Films

A careful biochemical analysis was conducted to compare the function and stability of the enzyme in its immobilized forms with respect to its free form. The Km value of the immobilized enzyme as well as the pH and temperature effects on its activity were tested at different times after immobilization (10, 30, and 100 days after the immobilization event).

**Figure 4:**
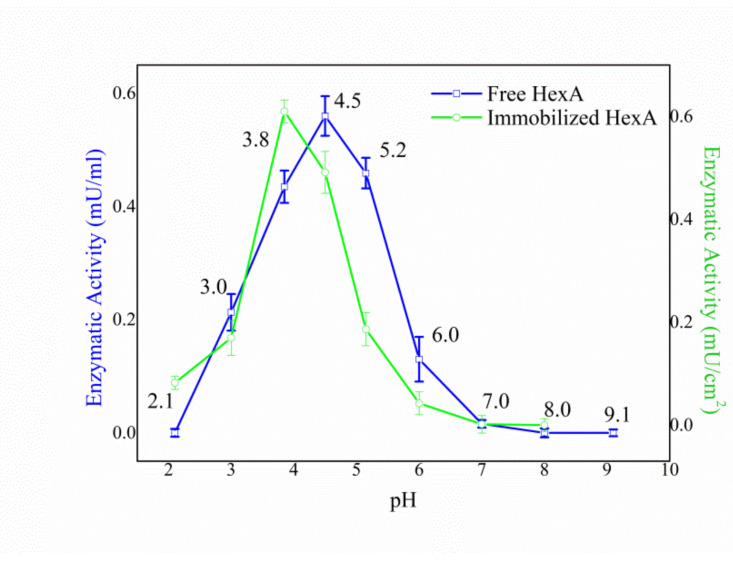
pH effect on the enzymatic activity of free (blue line) and immobilized (green line) HexA toward the artificial substrate MUG.

In the case of the pH dependence, analogous trends were recorded for immobilized HexA irrespectively of the aging of the HexA-PLA preparations. The values of the detections carried out 100 days after the immobilization event are reported as an example in Figure 4, together with the pH effect on HexA in its free form for comparison purposes.

Immobilization was found not to markedly alter the features of the protein: the enzyme in its immobilized forms, as its free form, maintains an optimum at acidic pH around a value of 4.0, characteristic of lysosomal hydrolases (Figure 4). Human lysosomal HexA is indeed known to show a pH optimum at 4.5, [63] in agreement with the value found for the recombinant HexA enzyme produced for this study.

**Figure 5:**
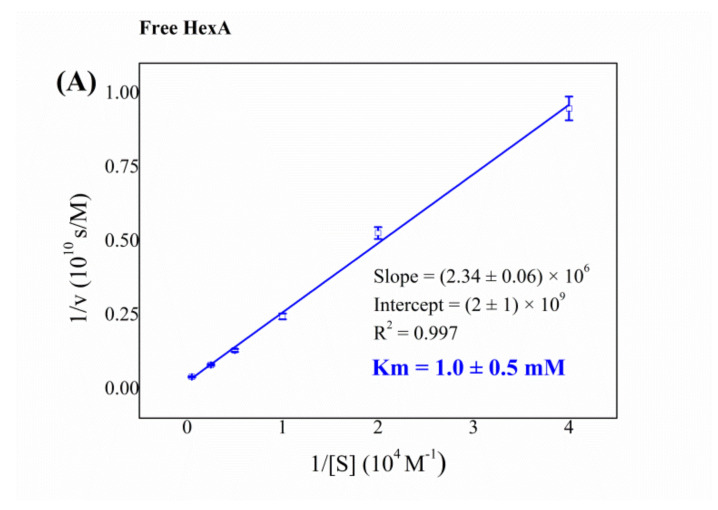

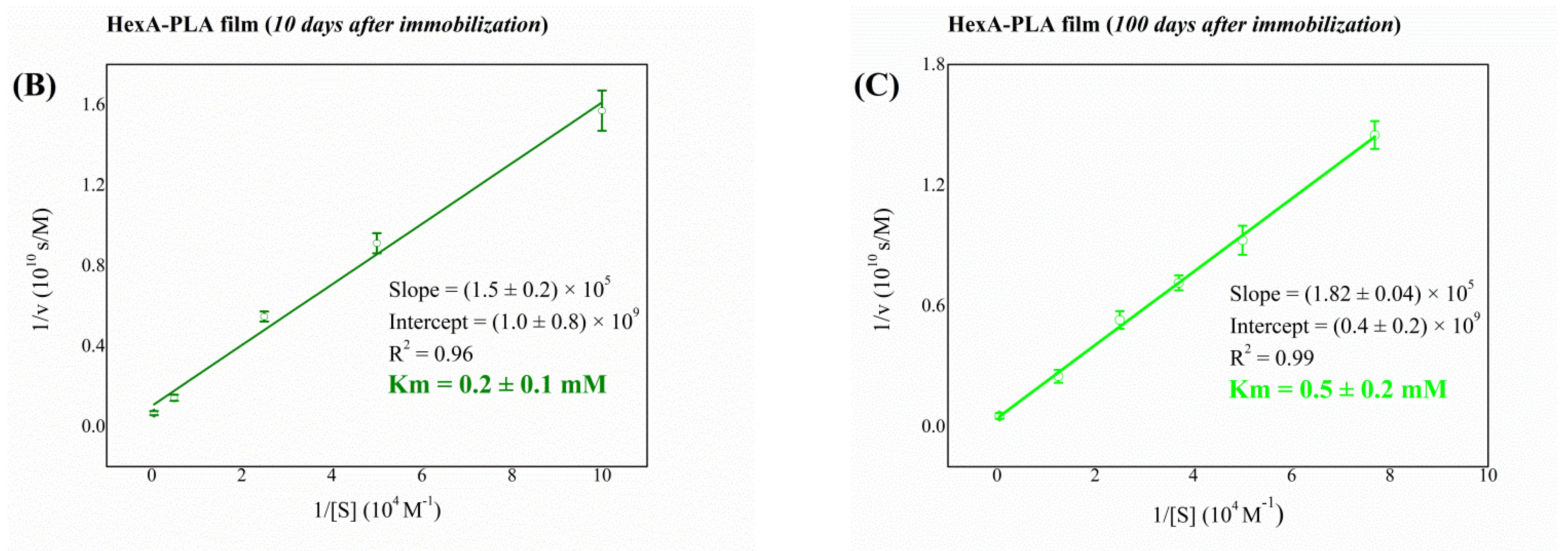
Km of free HexA (**A**) and immobilized HexA (lower panels) toward the artificial substrate MUG, 10 days (**B**), and 100 days (**C**) after the immobilization event.

Notable differences between free and immobilized HexA instead emerged from the Km analysis, carried out by using MUG as substrate (Figure 5 and Table 2). The enzyme kept high enzymatic affinity to its artificial substrate after bonding to the matrix, as is demonstrated by the lower Km values obtained for HexA-PLA film formulations, 0.2 mM and 0.5 mM recorded 10 and 100 days after immobilization, respectively, as opposed to a Km value of 1.0 mM, peculiar to the free enzyme. The latter value is in agreement with those reported in many previous studies where other human-derived HexA forms were studied against the same substrate [13,64,65]. This finding points to a greater affinity of the molecule towards MUG as a result of the immobilization process. However, the variations detected over time for the Km value of the HexA-PLA film formulations reveal an aging effect, with an alteration of the affinity of the immobilized enzyme toward its substrate.

**Figure 6:**
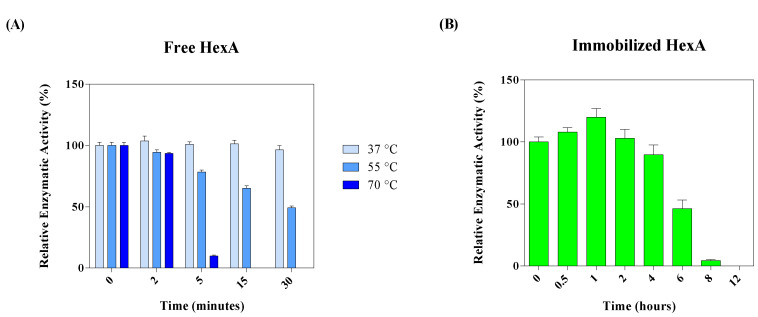
Thermal stability of free HexA (**A**) and immobilized HexA on PLA films (**B**). Results are reported as relative enzymatic activity (%) based on enzyme activity obtained at time 0.

As far as temperature effects are concerned, preliminary thermal stability studies have demonstrated that free HexA is a thermolabile molecule (as already reported in the literature) [63], as opposed to its immobilized form. Particularly, while free enzymatic molecules lose activity at high temperatures (50% activity reduction after 30 min at 55 °C and total loss at 70 °C, Figure 6A), immobilized HexA is exceptionally stable even at 70 °C. In fact, there are no sizeable changes in the activity of the immobilized enzymes after incubation at 70 °C up to 4-h-incubation time (Figure 6B). This is probably due to the enhanced stability provided by the cross-link of the enzyme on the PLA support, which prevents its denaturation with high temperatures.

**Figure 7:**
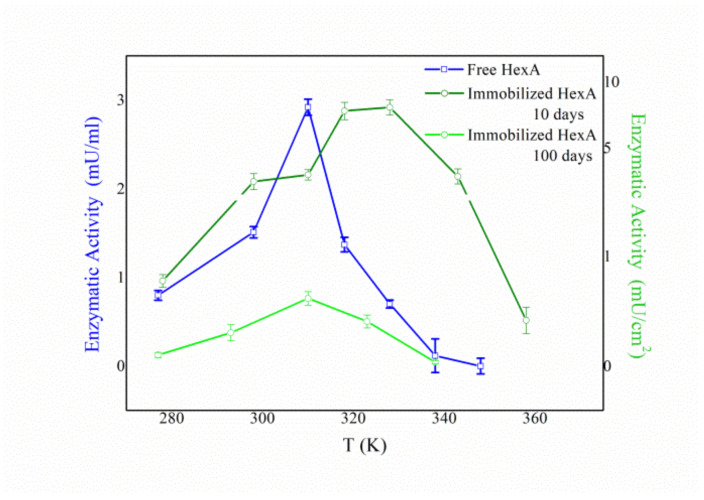
Temperature effect on the enzymatic activity of free HexA (blue line) and immobilized HexA on PLA films, 10 days (olive line), and 100 days (green line) after the immobilization event.

However, a peculiar aging effect was revealed for the enzymatic activity as a function of temperature as well (Figure 7). In fact, in the first days after the immobilization event, immobilized HexA was significantly active even at temperatures above the physiological one (olive line in Figure 7), with the temperature optimum shifting to 55 °C in the case of PLA films, as opposed to 37 °C typical of free HexA (blue line in Figure 7). The increased value of temperature optimum measured for the immobilized enzyme could indeed be related to the greater stability of the enzyme under high-temperature conditions. Conversely, at longer times after the immobilization (100 days), the temperature trend resembles that of the free enzyme (green line in Figure 7).

**Figure 8:**
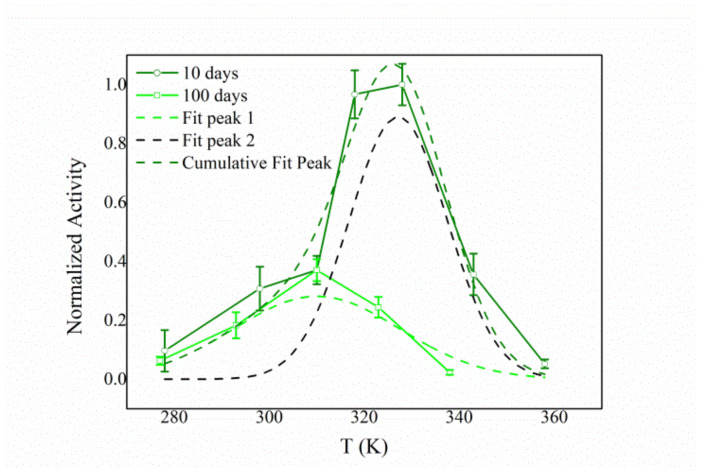
Temperature effect on the enzymatic activity of immobilized HexA on PLA films (10 days after the immobilization event) fitted by a double Gaussian function. The two peaks are assigned to two different forms of immobilized HexA.

This behavior could be rationalized by considering the possibility for the HexA enzyme to bind with the PLA film in different ways. Depending on the particular link between the protein and the film, the conformation and active site architecture of the former could be altered with respect to its free form, with direct consequences on its activity toward the substrate. In particular, the activity vs. temperature trend recorded 10 days after the immobilization (olive curve in Figure 8) can be fitted with a double Gaussian curve, one peaking at 37 °C (dashed green line) and the other peaking at about 55 °C (dashed black line). This finding could account for two different forms of immobilized HexA: one very much resembling its free form, and the other characterized by altered parameters. However, one hundred days after the immobilization, the temperature dependence changes and a simple curve peaking at 37 °C can be revealed (green line in Figure 7).

These results suggest that HexA can bind to the PLA film in two distinct ways, which are characterized by different stabilities over time. Specifically, one form of immobilized HexA, whose biochemistry is not significantly altered by the immobilization, exhibits an extremely long life exceeding one year; while a second form decays in about three months, which in any case represents a significant period of time for potential industrial and/or biomedical applications.

This explanation well agrees with the changes observed for the Km values as a function of aging, and with the trend of the enzymatic activity over time, where the initial decrease in activity can be ascribed to the reduced stability of the second form of immobilized HexA. Further to this, the changes in the MUG/MUGS activity ratio (Figure 3) can also provide a better understanding of this behavior. Starting from a value of 1.6, ascribed to enhanced activity of the α subunit obtained after immobilization, during the successive days, the ratio grows higher, peaking at a value of 2.4 after 38 days, meaning that the α subunit exhibits reduced stability over time. This ratio then is again reduced to a value of 1.2 after 100 days, supposedly as a consequence of the decay of the least stable of the two immobilized forms. After that time, the ratio grows back to the value of 2.4 as a result of the reduced activity of the α subunit, even in the other immobilized form. The different stability of the α and β subunits over time can also play a role in the changes measured for the Km value.

These results therefore lead the way for both industrial and biomedical applications of the HexA enzyme in its immobilized form on PLA supports.

## 4. Conclusions

This study demonstrates how the β-d-*N*-acetyl-hexosaminidase A enzyme in its immobilized form is extremely stable and potentially useful to develop novel biotechnological applications: in industry, because of its substantially increased stability towards heat, and especially in the medical field, where it could play a key role in the Enzyme Replacement Therapy (ERT) of LSDs. The enzyme was produced recombinantly and was subsequently obtained with a good degree of purification by resorting to established chromatographic techniques. The purified protein was then immobilized on PLA films, upon functionalization and activation of the material. The choice of this completely eco-friendly material makes the device biocompatible, an extremely important feature for applications in the biomedical field. Moreover, PLA can be easily shaped in order to design well-defined solid scaffolds, whose morphology is determined by the requirements of the final function they need to fulfill. The characteristics of the immobilized enzyme were tested through a series of biochemical investigations (evaluation of the Km value, as well as temperature and pH stability). Given the lower Km value than that of the enzyme in its free form, immobilized HexA was found to be more responsive to the synthetic substrate. Further to this, immobilization conveys great stability towards heat to the otherwise thermolabile HexA enzyme: immobilized HexA does not show any significant reduction in its activity after 4-h-incubation at temperatures as high as 70 °C. In addition, the investigation of the enzyme activity as a function of temperature revealed the presence of two distinct forms of immobilized HexA: one features a temperature optimum at a higher temperature than the physiological one, but its activity is reduced to zero beyond one hundred days; the other form exhibits unaltered biochemical parameters with respect to free HexA, with the enzymatic activity peaking at 37 °C, and proved to be extremely stable over time. In fact, the stability of the preparation as a function of passing time was thoroughly evaluated and, after the initial decrease trend due to deactivation of one of the two forms of immobilized HexA, its activity remained constant for a period exceeding one year without needing any particular treatment. The changes observed in the MUG/MUGS activity ratio over time provide further evidence of the presence of two distinct immobilized forms.

Starting from these encouraging initial data, enzyme-matrix systems could be developed for different biotechnological purposes: in particular, considerable interest lies in the industrial applications, as the enzyme maintains its activity even under extreme working conditions of high temperatures, and in the potentially viable biomedical application in the field of ERT, in order to effectively deliver a stable and active enzyme to the CNS, where it is most needed. In this light, specific HexA-PLA formulations, for example in the form of functionalized nanoparticles able to cross the BBB by virtue of their nanometric dimensions, can indeed be proposed as a new therapeutic approach for genetic LSDs such as Tay-Sachs and Sandhoff.

## 5. Patents

The above-described immobilization protocol is patent-pending (Italian Patent Application n. 102020000003344 filed on 19 February 2020).

## Figures and Tables

**Table 1:** Specific Activity towards MUG and purification ratio of HexA after affinity and ion-exchange chromatography.

Purification Step	HexA Specific Activity towards MUG (mU/mg)	Purification Ratio
Lysate HEK-HEXA	42.5	1
Affinity chromatography	185	4.4
Ion-exchange chromatography	1250	29.4

**Table 2:** Km values of free and immobilized HexA toward the artificial substrate MUG. Data concerning other human-derived HexA forms are taken from the literature.

System	Km (mM)
Free HexA from HEK-293 cells	1.0
Free HexA from HL-60 cells [13]	1.0
Free HexA from human placenta [64]	0.95
Free HexA from human liver [65]	0.8
Immobilized HexA (10 days after immobilization)	0.2
Immobilized HexA (100 days after immobilization)	0.5

## Data Availability

The data presented in this study are available on request from the corresponding author.
